# Cross–Species Transmission at the Wildlife–Livestock Interface: A Case Study of Epidemiological Inference From Mule Deer GPS Collar Data

**DOI:** 10.1002/ece3.71182

**Published:** 2025-04-10

**Authors:** Jennifer L. Malmberg, Jeremy Alder, Halcyon Killion, Danielle Buttke, Kim M. Pepin, George Wittemyer

**Affiliations:** ^1^ National Wildlife Research Center, Wildlife Services, Animal and Plant Health Inspection Service United States Department of Agriculture Fort Collins Colorado USA; ^2^ Department of Fish, Wildlife and Conservation Biology Colorado State University Fort Collins Colorado USA; ^3^ Wyoming State Veterinary Laboratory Laramie Wyoming USA; ^4^ Biological Resources Division National Park Service Fort Collins Colorado USA

**Keywords:** cross‐species transmission, emerging infectious disease, mule deer, *Mycoplasma bovis*, spillover, wildlife tracking, wildlife–livestock interface

## Abstract

In the current era of global change, the emergence of infectious diseases at the wildlife–livestock interface poses risks to biodiversity, agricultural economies, and public health. Driven by anthropogenic influence, increased sharing of resources between wildlife and livestock can promote cross‐species transmission, the consequences of which are challenging to predict. *Mycoplasma bovis* , an economically important bacterial pathogen in cattle, has recently emerged as a threat to other ungulate species. This study reports on a case of *M. bovis* in an intensively monitored free‐ranging mule deer ( *Odocoileus hemionus* ) in Colorado, USA, which presented an opportunity to describe the disease in a novel host and infer transmission and infection dynamics from GPS collar data. Following a mortality signal from a GPS‐collared adult female mule deer, field investigation revealed predation while postmortem examination further revealed severe, acute, fibrinosuppurative and necrotizing pleuropneumonia. Histopathological analysis, immunohistochemistry, and real‐time PCR confirmed *M. bovis* as the etiology. GPS collar data demonstrated spatial overlap with dairy cattle in the 50 days prior to death, implicating potential spillover from cattle as the transmission pathway. Reduced movement was identified 19 days prior to death, indicative of sickness behavior due to acute pneumonia. This case underscores the potential for *M. bovis* to cause severe disease in wild ungulates and highlights the value of thorough postmortem investigations as a routine component of studies involving wildlife tracking. The retrospective use of GPS collar data provides valuable insights into the movement ecology of wildlife exposed to novel pathogens, aiding in the understanding of cross‐species transmission and informing management strategies to reduce the potential for spillover.

## Graphical abstract

*Mycoplasma bovis* is bacterial pathogen of cattle that is rarely detected in wildlife. Our intensive monitoring of free‐ranging mule deer led to the discovery of *Mycoplasma bovis* as the cause of death in an animal that had recently selected for use of a dairy premise. Our findings highlight the potential for spillover inference from intensively monitored wildlife, while also contributing foundational knowledge about a specific emerging infectious disease at the wildlife‐livestock interface.

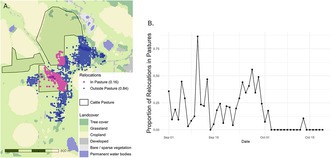

## 1 Introduction

Coexistence between wildlife and livestock is central to the maintenance of healthy, sustainable agroecosystems (Barroso and Gortázar 2024). However, the overlap of domestic and free‐ranging species often presents ample opportunity for infectious disease transmission that either adversely impacts biodiversity or jeopardizes livestock production and trade (Miller et al. 2013). Notable examples of diseases maintained in wildlife reservoirs include brucellosis (Cross et al. 2010) and bovine tuberculosis (Meunier et al. 2017), both of which pose an economic threat to the cattle industry while also carrying a public health risk. Counter to economic threats, some pathogens primarily carry ecological consequences for free‐ranging hosts. Bighorn sheep pneumonia, for example, is a population‐limiting polymicrobial disease of major conservation concern for which domestic caprids serve as subclinical reservoirs (Cassirer et al. 2018; Besser et al. 2013). These classic examples of wildlife–livestock conflicts are well studied and well characterized but remain challenging to manage and mitigate. As land use and climate continue to change, new conflicts between wildlife and livestock are expected to materialize, including the emergence of infectious diseases in new host species or new geographic regions (Baker et al. 2022; Gottdenker et al. 2014).

Recently, cross‐species transmission of rapidly evolving RNA viruses has been the subject of much important scientific research (Feng et al. 2023; Caserta et al. 2024). In addition to viruses, the remarkable evolutionary capacity of microbes extends to some lesser known pathogens that evolve through unique mechanisms. Mycoplasmas are the smallest self‐replicating organisms described to date. They are ubiquitous, cell wall‐less bacteria belonging to the class Mollicutes and have evolved through genetic reduction, parsing down the genome to only essential elements and relying on host enzymes and proteins for vital functions (Caswell and Archambault 2007; Citti and Blanchard 2013; Maunsell and Donovan 2009). Mycoplasmas have historically been characterized as host‐specific (Citti and Blanchard 2013), with each Mycoplasma species infecting a narrow range of predictable taxa (e.g., *Mycoplasma canis* in canids, *Mycoplasma ovipneumoniae* in caprids, and *Mycoplasma pneumoniae* in humans). More recently, however, a broader, more complex picture of host range has emerged through detections of mycoplasmas in atypical species (Highland et al. 2018; Malmberg et al. 2020). In novel hosts, mycoplasmas are often associated with increased virulence (Caswell and Archambault 2007). For example, spillover of *Mycoplasma gallisepticum* from domestic poultry to house finches ( *Haemorhous mexicanus* ) in the mid‐1990s resulted in rapid population reductions (Ley et al. 1996, 2006). Similarly, the emergence of *Mycoplasma aggassizzi* in the 1980s led to major declines of the Mojave desert tortoise ( *Gopherus agassizii* ) resulting in threatened species status (Brown et al. 1994).

*Mycoplasma bovis* is one of several mycoplasma species infecting cattle. In beef cattle, *M. bovis* causes respiratory disease and polyarthritis in feedlot calves, whereas mastitis is the most common presentation in dairy cattle (Maunsell and Donovan 2009; Maunsell et al. 2011). In the early 2000s, *M. bovis* emerged in ranched bison ( *Bison bison* ) as a highly transmissible respiratory pathogen with a high case fatality rate (Janardhan et al. 2010; Dyer et al. 2008, 2013). In 2019, *M. bovis* was first reported as the cause of a large die‐off of pronghorn ( *Antilocapra americana* ) in Wyoming, USA (Malmberg et al. 2020). Ongoing and increasing losses in bison (Schwartz et al. 2024) and subsequent *M. bovis* outbreaks in pronghorn (Janardhan et al. 2010) have recently prompted concerns for the economic and ecological impacts of *M. bovis* to ungulate species more broadly. Despite this increasing prevalence and significance, little is known about where these cross‐species spillover events originated nor how to prevent future spillover transmission events.

Mycoplasmosis is not commonly identified or reported in cervids. To the best of our knowledge, there is only one detailed report of *M. bovis* in cervids, which describes an outbreak in captive white‐tailed deer ( *Odocoileus virginianus* ) characterized by acutely fatal respiratory disease (Dyer et al. 2004). The National Center for Biotechnology Information hosts a single *M. bovis* isolate from a free‐ranging white‐tailed deer and a single isolate from a mule deer ( *Odocoileus hemionus* ) (Register et al. 2019), indicating the susceptibility of free‐ranging cervids. However, characterization and further documentation of this disease in deer is absent from the literature. Following the outbreak of *M. bovis* in pronghorn in Wyoming, opportunistic surveillance for *M. bovis* in sympatric deer did not reveal any active infections (Johnson et al. 2022). Here, we report on a case of *M. bovis* in a free‐ranging adult female mule deer and characterize the pathology, movement ecology, and spatial overlap with cattle prior to death according to GPS collar data.

## 2 Methods

### 2.1 Study Area and Mule Deer Collaring

This female mule deer was captured as part of a national, multi‐investigator study that aimed to implement broad‐scale, targeted surveillance for SARS‐CoV‐2 in deer to better understand human–deer interactions as risk factors for virus presence and persistence in deer populations across the United States (Pepin et al., in review). The Colorado study region is located in Larimer and Weld counties, with deer captured on parcels of private land in peri‐urban habitat. Two rivers bisect the study region, which is characterized by short grass prairie ecosystems interspersed with suburban development and livestock production facilities, including dairies and feedlots. National landcover database (NLCD) habitat types include woody wetlands, emergent herbaceous wetlands, cultivated crops, and grassland or herbaceous vegetation.

In April of 2023, deer were captured either by darting or Clover trap, anesthetized by intramuscular injection with BAM (Butorphanol 0.6 mg/kg, Azaperone 0.2 mg/kg, and Medetomidine 0.2 mg/kg), and equipped with a solar powered GPS tracking collar (Savannah Tracking LTD, Kilifi, Kenya). All capture, handling, and sampling protocols were approved by the Colorado State University Animal Care and Use Committee (IACUC protocol #3872) and capture and sampling collection were permitted under the Colorado Parks and Wildlife Scientific Collection Permit (License # 3033484170). The GPS collars used in this study record geographic coordinates (latitude and longitude) every 15 min to allow for detailed monitoring of the deer's movements. At the time of the collaring, the adult female mule deer on which this study is focused was in apparently good health, weighing 74.3 kg.

### 2.2 Postmortem Investigation

Though not formally a component of the national SARS‐CoV‐2 study, mortality investigations were performed on collared deer when feasible. Necropsy methods and the extent of postmortem diagnostics varied greatly across SARS‐CoV‐2 study sites. For our Colorado study region, we retrieved carcasses and performed necropsies at the National Wildlife Research Center. On October 20, 2023, we received a mortality signal for a GPS‐collared mule deer located north of Fort Collins, Colorado, USA, in Larimer County. We investigated the mortality and identified a mountain lion ( *Puma concolor* ) at the carcass along with a drag trail. The carcass was partially consumed and partially cached. The head and the cranial half of the carcass remained intact, while much of the caudal half of the carcass was absent. Puncture wounds with subcutaneous hemorrhage were present on the rostrum, implicating predation as the cause of death. We retrieved the carcass and transported it to the National Wildlife Research Center for postmortem examination.

### 2.3 Necropsy and Histopathology

Our research team included a board‐certified veterinary pathologist who performed necropsy to establish the cause of death. Necropsy included gross examination and collection of available tissues, which in this case were limited due to predation and included lung, heart, skeletal muscle, tongue, bone marrow, brain, and retropharyngeal lymph node. For histopathology, tissues were fixed in 10% neutral buffered formalin and processed conventionally before embedding in paraffin wax. Sections cut at 5 μm were stained with hematoxylin and eosin.

### 2.4 SARS‐CoV‐2 Testing

We performed testing for SARS‐CoV‐2 at the National Wildlife Research Center in Fort Collins, Colorado. Serum was tested for antibodies according to Chandler et al. (2021). Oral and nasal swabs were tested for SARS‐CoV‐2 RNA as described in Feng et al. (2023).

### 2.5 Ancillary Diagnostics

We performed ancillary diagnostics at the discretion of the pathologist to maximize confidence in the cause of death and assess for comorbidities or predisposing factors. In this case, we performed multiple assays to confirm a suspected diagnosis of *M. bovis* , to rule out other causes of respiratory disease, and to routinely establish postmortem chronic wasting disease (CWD) status of deer in this CWD‐endemic region. We performed all ancillary diagnostics at Wyoming State Veterinary Laboratory (WSVL) unless otherwise specified.

#### 2.5.1 *Mycoplasma bovis* Real‐Time PCR

We extracted DNA from lung using the DNeasy Blood & Tissue Kit (Qiagen, Germantown, Maryland, USA) and performed real‐time quantitative PCR (qPCR) targeting the *oppD* gene using primers and probes described in Loy et al. (2018), cycle conditions described in Johnson et al. (2022) and a cycle threshold of ≤ 35 for positivity.

#### 2.5.2 Mycoplasma Culture

We placed approximately 20 mg of lung tissue in 4 mL of Mycoplasma enrichment broth (mycoplasma broth, Hardy Diagnostics, Santa Maria, California, USA) and incubated with a loose lid at 37 C in 10% CO_2_ for 72 h. Following incubation, we used a sterile swab to evenly spread 100 μL of broth onto a commercial Mycoplasma spp. agar (mycoplasma agar with cefoperazone, Hardy Diagnostics) plate, which was then incubated at 37 C in 10% CO_2_ until a confluent layer of colonies was present across the plate.

#### 2.5.3 *Mycoplasma bovis* Immunohistochemistry

We performed immunohistochemistry (IHC) for *M. bovis* on lung tissue derived from the same blocks as those stained with hematoxylin and eosin, as described in Malmberg et al. (2020).

#### 2.5.4 Bovine Respiratory Disease Panel

To rule out common respiratory viruses of domestic ruminants, we performed a bovine respiratory panel, which includes PCR for bovine herpesvirus‐1, bovine viral diarrhea virus, parainfluenza‐3 virus, and bovine respiratory syncytial virus as described in Malmberg et al. (2020).

#### 2.5.5 Acid‐Fast Stain

To exclude the possibility of mycobacterial infection, we performed an acid‐fast stain on the lung as described in Malmberg et al. (2020).

#### 2.5.6 Aerobic Culture

To exclude the possibility of bacterial co‐infection, we performed aerobic culture on lung tissue as described in Malmberg et al. (2020).

#### 2.5.7 Chronic Wasting Disease ELISA

To establish postmortem CWD status, enzyme‐linked immunosorbent assay was performed on medial retropharyngeal lymph node tissue at the Wyoming Game and Fish Wildlife Health Laboratory as described in Hibler et al. (2003).

### 2.6 Deer–Cattle Interaction Analysis

We used ArcGIS Pro 3.3 to plot the deer's GPS locations from the last 50 days of her life (September 1st to October 19th) relative to cattle pastures in the study system. We generated static polygons representing cattle‐occupied pastures based on information provided by the local dairy management. Cattle pastures consisted of grassland areas designated for grazing and were confirmed to contain cattle throughout the entire 50‐day sampling period. The deer's GPS locations were intersected with these polygons to identify times and locations of potential cattle–deer interactions, given finer resolution information on cattle locations was not available.

We used a 50‐day time window in our analysis based on the pathologist's derived estimate of the duration of disease. We calculated the proportion of GPS points that intersected the cattle‐occupied polygons, daily step distance, and net square displacement (NSD) for each point in the 50‐day period. Daily step distance, calculated by summing the Euclidean distances between consecutive GPS locations for each day, was chosen to capture overall mobility and potential changes in daily movement effort. NSD, calculated as the squared Euclidean distance of each GPS location from the mortality site, was selected to assess spatial behavior, including shifts from wide‐ranging movements to more localized activity around the location of death. These metrics were plotted over time to identify any changes in movement behavior that could indicate the onset of clinical disease. A significant drop in daily step distance or NSD was hypothesized to reflect reduced mobility and disease progression, consistent with clinical signs of *M. bovis* , such as respiratory distress.

## 3 Results

### 3.1 Gross Pathology

**FIGURE 1:**
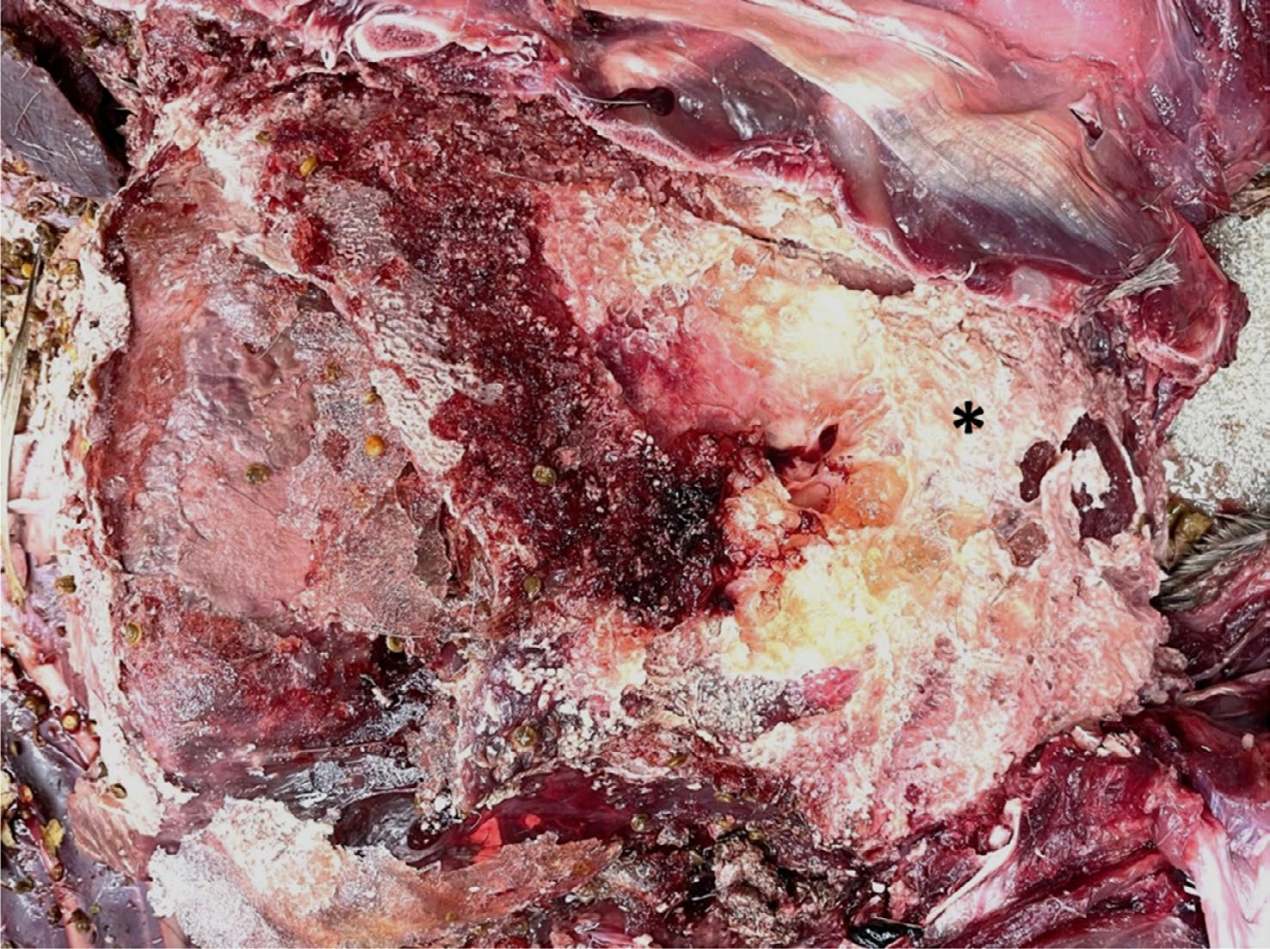
Mule deer ( *Odocoileus hemionus* ) thoracic cavity: *Mycoplasma bovis* is grossly characterized by abundant fibrin (asterisk) covering the lungs and expanding the pleura. Lung lobes are consolidated, particularly in the cranioventral region, with discrete to coalescing foci of acute, suppurative inflammation and necrosis.

The carcass was in good postmortem condition with mild autolysis and partial consumption/scavenging of the abdominal viscera and hindlimbs. The animal was in healthy body condition (BCS 3/5) with ample visceral and subcutaneous adipose tissue. The bone marrow was pale pink, solid, and opaque. There were three deer keds ( *Lipoptena cervi* ) and four to six hard ticks (*Dermacentor* spp.), the majority of which were found on the head and neck region. There were two bot fly larvae (*Cephenemyia* spp.) within the nasopharynx. There were multiple puncture wounds associated with variable degrees of acute hemorrhage that often extended through the subcutis and deep into the underlying skeletal muscle. Penetrating wounds were most extensive on the muzzle and at the base of the left ear. Punctures measured ~0.5 cm in diameter, with ~3.0 cm between paired wounds. The thorax contained approximately 200 mL of serosanguineous fluid. The pleura was diffusely covered by thick mats of yellow fibrin (Figure 1). Subjacent to the fibrin, lung tissue was consolidated and discolored dark purple to pale tan, with pale foci extending deep into cut section. Greater than 70% of the lung tissue was affected. The reproductive tract was missing due to predation/scavenging, along with the abdominal viscera. The proximate cause of death was determined to be severe, acute, fibrinosuppurative to necrotizing pleuropneumonia suggestive of pulmonary mycoplasmosis (Malmberg et al. 2020), while mountain lion predation was assigned as the immediate cause of death based on gross lesions and field investigation. Our top differentials for the pneumonia included *Trueperella pyogenes* , *Mycobacterium* spp., and other bacterial etiologies, possibly with viral co‐infecting agents.

### 3.2 Histopathology

**FIGURE 2:**
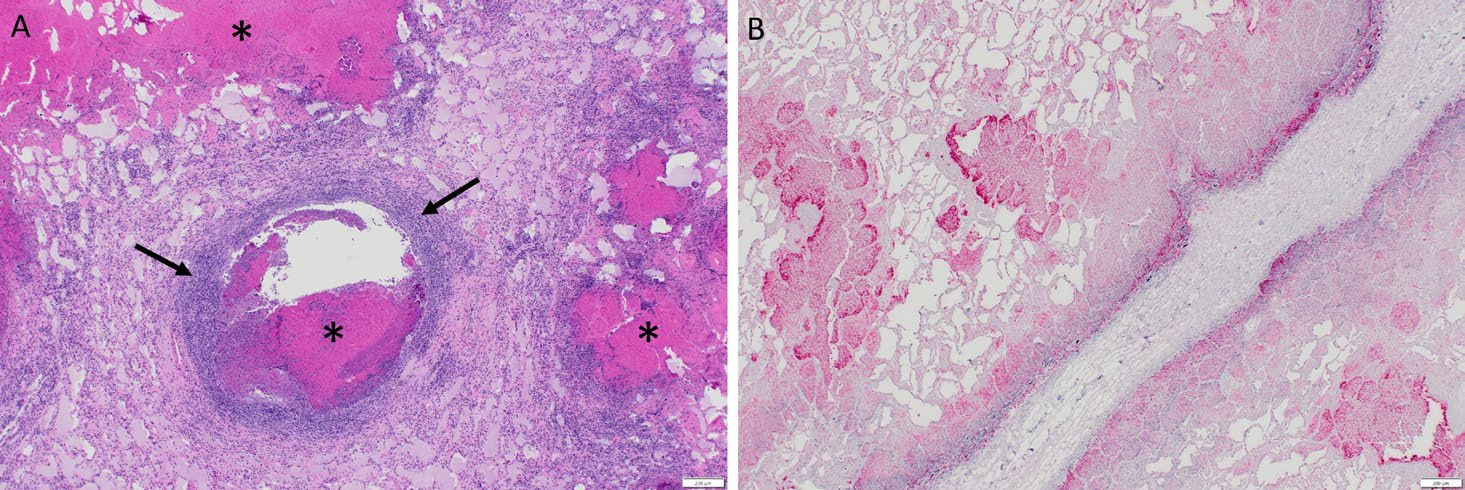
(A) Histologic lesions of *Mycoplasma bovis* in a mule deer ( *Odocoileus hemionus* ) are characterized by foci of coagulative necrosis (*) scattered throughout the lungs, which are surrounded by infiltrating neutrophils. Small airways are filled with edema and fibrin. (B) Immunohistochemistry performed on lung tissue from a mule deer ( *Odocoileus hemionus* ) reveals positive staining for *Mycoplasma bovis* within and adjacent to necrosuppurative lesions. Immunostaining is strongest at the edges of the necrotic foci.

Histopathologic examination of lung tissue revealed large, coalescing foci of acute necrosis affecting > 70% of the tissue obtained from the cranioventral lungs. Foci of necrosis were surrounded by peripheral aggregates of degenerate neutrophils admixed with fewer lymphocytes and plasma cells (Figure 2A). Alveoli were characterized by septal necrosis and were filled with edema and fibrin. There was expansion of the pleura with fibrin, greater than 10 times the normal thickness. No evidence of comorbidities was identified histologically.

### 3.3 *Mycoplasma bovis* Testing

Immunohistochemical evaluation for *M. bovis* revealed abundant positive staining in the previously described lung lesions, with the intensity of immunoreactivity increasing toward the periphery of the necrotic foci (Figure 2B). Real‐time PCR for *M. bovis* was positive, with a cycle threshold value of 17.46. Lung tissue was also culture positive.

### 3.4 SARS‐CoV‐2 Testing

SARS‐CoV‐2 antibodies were not detected by surrogate virus neutralization testing, and SARS‐CoV‐2 RNA was not detected by qRT‐PCR.

### 3.5 Ancillary Diagnostics

Chronic wasting disease was not detected by ELISA. Respiratory viruses (bovine herpesvirus‐1, bovine respiratory syncytial virus, bovine viral diarrhea virus, and parainfluenza‐3 virus) were not detected by PCR. Aerobic culture did not identify co‐infecting bacterial pathogens. No acid‐fast bacteria were identified in the lung using an acid‐fast stain.

### 3.6 Movement and Interaction Analyses

**FIGURE 3:**
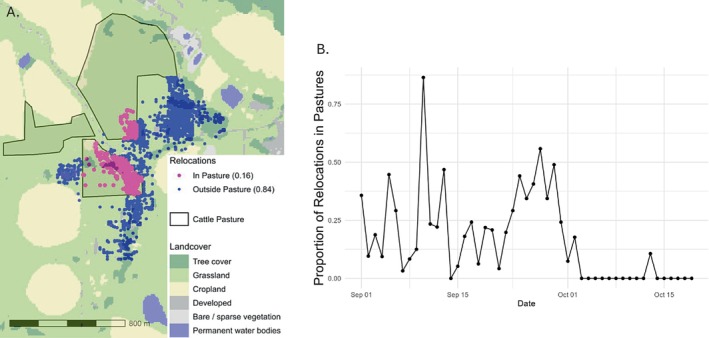
(A) GPS collar locations of an adult female mule deer ( *Odocoileus hemionus* ) within 50 days of death. Coordinates were overlaid with cattle‐use polygons. Within 50 days prior to death caused by *Mycoplasma bovis* , 16.5% of coordinates intersected polygons representing pastures actively inhabited by dairy cattle. (B) Daily proportion of mule deer locations that intersected cattle pasture within 50 days of death. Cattle pasture was regularly visited ~7–4 weeks prior to death, during which time there was notable daily variation. In late September about 4–3.5 weeks prior to death, daily variation decreased with the proportion of time spent in cattle pasture remaining above 25% for about a week. Minimal overlap with cattle was noted in the final 2–3 weeks of life.

**FIGURE 4:**
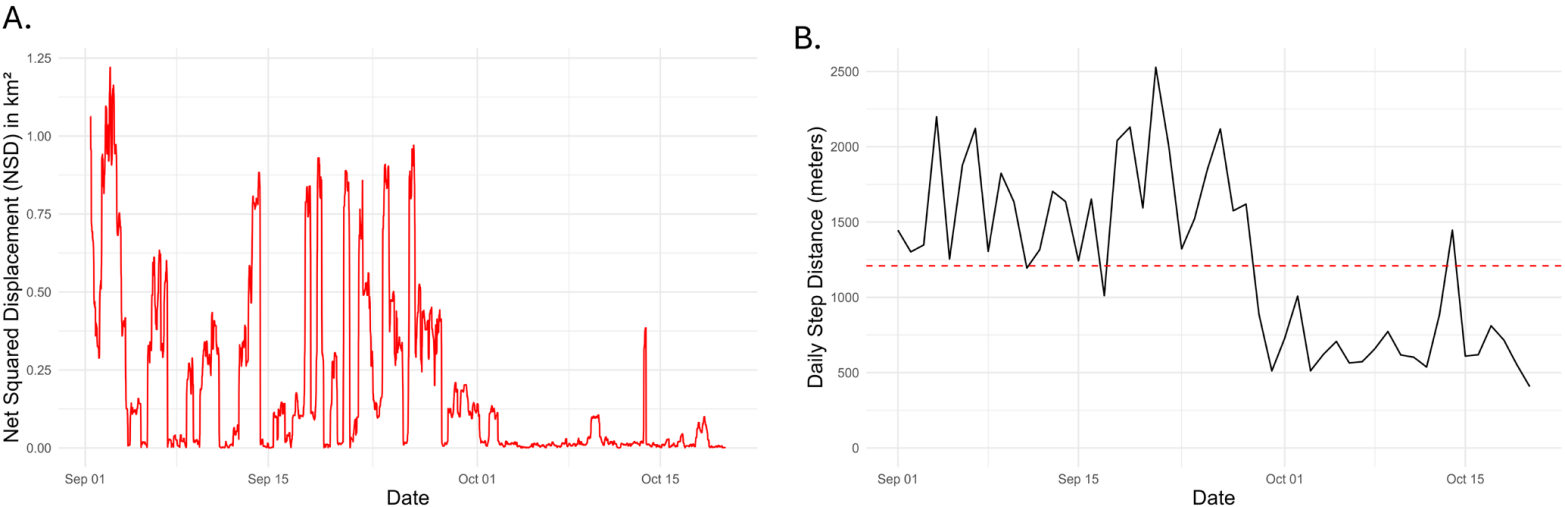
(A) Net squared displacement (NSD) of the last 50 days of life of an adult female mule deer ( *Odocoileus hemionus* ), calculated as the squared distance of each GPS point to the deer's mortality location). (B) Daily, cumulative step distance for the last 50 days of the deer's life. Both metrics of movement decreased in the final 3 weeks of the deer's life.

Approximately 16.5% of GPS points recorded during the 50 days prior to mortality intersected with occupied cattle pastures (83.5% were recorded outside; Figure 3A). Peak deer–pasture overlap occurred between 3 and 6 weeks prior to death (Figure 3B), during which the deer spent an average of 29% of the day within cattle pastures. The deer showed minimal use of cattle pastures in the final 2 weeks of life. The average daily step distance during the 50‐day period was 1234.5 m (Figure 4A). From 50 to 20 days prior to death, the average daily step distance was 1592.2 m. On October 1st (19 days prior to death), daily step distance declined dramatically, and in the final 19 days, average daily step distance was 712 m, representing a 55.2% reduction from the previous 31 days. Similarly, net squared displacement (Figure 4B) demonstrated relatively wide‐ranging movements up to October 1st, with an average NSD of 0.300 km^2^ per GPS point. After October 1st, deer space use was localized within a small area until death, with an average NSD of 0.0249 km^2^ per GPS point, 91.7% of the pre‐October 1 average.

## 4 Discussion

The lurking threat of pathogen spillover from reservoir hosts to novel species is pervasive yet difficult to depict or quantify. Many spillover events are silent, going unnoticed due to a lack of sustained transmission in the novel host (Parrish et al. 2008). In wildlife especially, spillover events have often reached epizootic status by the time they are identified (Judson et al. 2016). Given the rarity of early recognition, the identification of an isolated case of spillover in wildlife presents a valuable opportunity to characterize the pathology, which can inform future surveillance efforts. For the mule deer described in this study, *M. bovis* was the top differential for the pneumonia based on the experience of the pathologist, as the gross lesions closely resembled those seen with epizootic *M. bovis* in pronghorn (Malmberg et al. 2020; Johnson et al. 2022). Specifically, the severe, acute fibrinous pleuropneumonia with coalescing foci of necrosis is characteristic of *M. bovis* in pronghorn and similar to the severe presentation of this agent in bison (Janardhan et al. 2010). This striking similarity of lesions across several ungulate species is indicative of high virulence in naïve hosts, in stark contrast to the smoldering, chronic disease that is typical in the bovine reservoir. The confirmation of *M. bovis* in a mule deer also lends support to growing evidence of a broader *M. bovis* host range than previously recognized. Accordingly, *M. bovis* should be a differential for pneumonia in mule deer and other species of free‐ranging ungulates, especially those that overlap with cattle.

The ability to trace back animal movements to infer transmission pathways and infection dynamics following identification of spillover to wildlife is rare. Our discovery of *M. bovis* in an intensively monitored mule deer presented a unique opportunity to examine habitat selection and fine scale movement ecology prior to death. We hypothesized that the onset of clinical signs would be detectable as a distinct reduction in daily step distance followed by localization of activity within the vicinity of the mortality site. To capture this shift in movement, we selected 50 days prior to death as an appropriate time window based on the acute nature of the pneumonia, the body condition of the deer, and previous characterization of *M. bovis* in other wildlife species. While *M. bovis* has not been previously described in mule deer, acute pneumonia is characteristic of cases described in pronghorn (Malmberg et al. 2020), white‐tailed deer (Dyer et al. 2004), and bison (Janardhan et al. 2010; Dyer et al. 2008). In this mule deer, the lung lesions lacked features of chronicity such as interstitial fibrosis or dystrophic mineralization within foci of necrosis, and visceral stores of adipose tissue were ample, providing further evidence that this animal was recently in good health. Supporting our hypothesis, we identified a distinct change in behavior beginning 19 days prior to death, measured as a decrease in daily step distance and NSD from the mortality site, which we can infer corresponds to the onset and progression of clinical disease.

Our findings indicate use of an active dairy cattle pasture prior to the onset of sickness behavior (Figure 3A), suggesting dairy cattle as the source of cross‐species transmission. While we could not confirm close contact with infected cattle, we show spatiotemporal overlap with an established reservoir host (Figure 3B) 3–6 weeks prior to detection of a novel pathogen in a free‐ranging animal. Detection of *M. bovis* in wild ungulates is uncommon, and attraction to livestock resources has been hypothesized as a potential driver of *M. bovis* emergence in free‐ranging pronghorn (Johnson et al. 2022). This study therefore provides further evidence of *M. bovis* spillover to wildlife from livestock and suggests that such events may occur more commonly than recognized.

The potential for intraspecific transmission of *M. bovis* in mule deer remains unclear. We were unable to thoroughly assess for similar mortalities in sympatric deer that were not a part of this study and did not identify *M. bovis* in other mule deer monitored for the SARS‐CoV2 study. Previous studies did not detect *M. bovis* in apparently healthy mule deer in regions of recent epizootics in pronghorn, indicating that subclinical cases are unlikely (Johnson et al. 2022). Our findings indicate that mule deer are susceptible to *M. bovis* , which presents as severe, acute pneumonia resulting in sickness behavior that may limit intraspecific transmission. If, however, mule deer contact rates were to remain high following spillover, for example, due to congregation around livestock resources or supplemental feed, the potential for *M. bovis* epizootics would be of significant concern, particularly for suppressed populations already vulnerable to CWD, habitat degradation, high harvest pressure, or other sources of additive mortality. Thus, our documentation of *M. bovis* in a mule deer provides rationale for discouraging practices that congregate deer on the landscape.

Mountain lion predation was assigned as the cause of death based on field investigation of this mortality. A mountain lion was present at the site when the collar was recovered and the carcass retrieved, and predation was confirmed by a board‐certified pathologist based on puncture wounds with subcutaneous hemorrhage. This study therefore highlights the value of thorough cause‐specific mortality investigations as a routine part of wildlife research, as we were surprised to confirm *M. bovis* as the proximate cause of death of a deer that appeared to be a straightforward case of predation at first glance. Infectious diseases, however, can predispose animals to predation (Miller et al. 2008), and the extent to which predators keep herds healthy by removing sick individuals is an area of increasing interest (Wild et al. 2011; Brandell et al. 2022; Lopez et al. 2023), providing further rationale for detailed postmortem examinations of prey species. While not a particular concern of ours in this case given that reports of *M. bovis* are limited to ungulate species, consumption of prey infected with novel pathogens can also present opportunities for further spillover through intermediate hosts, which can promote pathogen adaptation and evolution (Cleaveland et al. 2001; Malmberg et al. 2021).

While we could not pinpoint the time of exposure or confirm direct contact with livestock, our study of high fix rate collar data permits inference of spillover source and establishes a general timeline for the clinical stage of an infectious disease in a novel host. A limitation of this study is that we did not perform surveillance for *M. bovis* in local dairy cattle. However, *M. bovis* is ubiquitous in cattle (Szacawa et al. 2016), and while generally labile outside the host, environmental survival of *M. bovis* has been documented up to 8 months in recycled sand bedding from dairy farms (Justice‐Allen et al. 2010). Future studies could include local or regional targeted surveillance for *M. bovis* with sequencing of isolates and phylogenetic analysis as an additional line of evidence for the inferred transmission pathway. Observations of mule deer interactions with livestock may also be of value, especially to elucidate mule deer attractants that could be targeted by management actions.

Advances in wildlife tracking technology have enhanced our ability to monitor animal movements, facilitating assessment of fine‐scale behaviors and dramatically increasing the amount of available GPS data (Abrahms et al. 2021; Hertel et al. 2020; Nathan et al. 2022). While these advances are commonly leveraged in ecological research, they are rarely leveraged in epidemiological studies. Recently, GPS collar data was used to infer sickness syndromes in mule deer with chronic wasting disease (CWD) (Barrile et al. 2024), indicating that measurable changes in movement can be indicative of clinical signs of endemic diseases. The current study suggests that changes in the movement ecology of intensively monitored animals could also cue researchers in on instances of cross‐species transmission, underscoring the value of thorough mortality investigations even when the cause of death appears obvious based on field observations. Studies of monitored wildlife would therefore benefit from additional funding to specifically support mortality investigations, including timely field response, examination by veterinary pathologists, and ancillary diagnostics.

The wildlife–livestock interface presents ample opportunities for cross‐species tranmission (Miller et al. 2013). Spillover events are often difficult to discern in wildlife, but early recognition can inform the risk of more extensive outbreaks, which can guide management and potentially abate the ecological impacts of emerging infectious diseases. This study indicates that characteristic lung lesions can provide a cue for biologists and others to pursue *M. bovis* testing of wild ungulates, while also highlighting the more general utility of GPS collar data for spillover inference, particularly for cryptic exposures originating from known reservoir hosts. While little is known about *M. bovis* in deer at this time, opportunistic surveillance could elucidate possible management actions, such as mitigation of deer attractions on dairy pastures or deterrence during high‐risk periods or seasons. Recent spillover events (e.g., SARS‐CoV‐2 in deer; Chandler et al. 2021; Hale et al. 2022), highly pathogenic avian influenza virus in dairy cattle (Caserta et al. 2024) highlight a growing intersection between the fields of wildlife management, disease ecology, and public health, calling for new tools to monitor for cross‐species transmission events prior to the emergence of enzootics and epidemics. Intensive tracking of wildlife has increased in response, which we show can be used not only to better understand interspecific interactions, but also to monitor unusual behaviors and thoroughly investigate wildlife mortalities and the clinical course of diseases in novel hosts.

## Author Contributions

**Jennifer L. Malmberg:** conceptualization (lead), formal analysis (equal), investigation (lead), methodology (equal), project administration (lead), writing – original draft (lead), writing – review and editing (equal). **Jeremy Alder:** data curation (equal), formal analysis (equal), investigation (equal), writing – original draft (supporting), writing – review and editing (supporting). **Halcyon Killion:** formal analysis (equal), investigation (supporting), methodology (equal), writing – original draft (supporting), writing – review and editing (supporting). **Danielle Buttke:** conceptualization (supporting), investigation (supporting), writing – original draft (supporting), writing – review and editing (equal). **Kim M. Pepin:** conceptualization (supporting), funding acquisition (lead), project administration (equal), writing – original draft (supporting), writing – review and editing (equal). **George Wittemyer:** conceptualization (equal), formal analysis (equal), investigation (equal), project administration (equal), writing – original draft (supporting), writing – review and editing (equal).

## Disclosure

The authors were either engaged at the project initiation phase, the diagnostic phase, and/or in the analysis and manuscript preparation that followed the diagnosis. Several scientific disciplines are represented in the authorship, and each author contributed unique expertise.

## Conflicts of Interest

The authors declare no conflicts of interest.

## Supporting information


**Table S1.** Diagnostic assays performed on mule deer ( *Odocoileus hemionus* ) tissues, serum, and swabs.

## Data Availability

Diagnostic data are summarized in Table S1. Deer locations cannot be made available because mule deer are a hunted species.
