# Supplementary material for: Cross–Species Transmission at the Wildlife–Livestock Interface: A Case Study of Epidemiological Inference From Mule Deer GPS Collar Data
Source: Ecol Evol. 2025 Apr 10;15(4):e71182. doi: 10.1002/ece3.71182 (PMC11985357; doi:10.1002/ece3.71182)
Supplement: Supplementary file 1 — Table S1. Diagnostic assays performed on mule deer ( Odocoileus hemionus ) tissues, serum, and swabs. [file ECE3-15-e71182-s001.docx]

**Supplemental Table 1**. Diagnostic assays performed on mule deer (*Odocoileus hemionus*) tissues, serum, and swabs.

| **Diagnostic assay** | **Result** | **Sample tested** | **Laboratory** |
| --- | --- | --- | --- |
| *Mycoplasma bovis* PCR | Detected (Cycle threshold 17.46) | Lung | Wyoming State Veterinary Laboratory |
| Mycoplasma culture | Positive | Lung | Wyoming State Veterinary Laboratory |
| *Mycoplasma bovis* immunohistochemistry | Positive | Lung | Wyoming State Veterinary Laboratory |
| SARS-CoV-2 surrogate virus neutralization | Antibodies not detected | Serum | National Wildlife Research Center |
| SARS-CoV-2 quantitative reverse-transcriptase PCR | Viral RNA not detected | Oral and nasal swabs | National Wildlife Research Center |
| Gross pathology and histopathology | Severe, acute, fibrinosuppurative and necrotizing pleuropneumonia; no comorbidities detected | Multiple tissues | National Wildlife Research Center |
